# Case Report: Hemophagocytic lymphohistiocytosis associated with NBAS gene variant and Epstein-Barr virus (EBV) infection

**DOI:** 10.3389/fped.2025.1635534

**Published:** 2025-08-14

**Authors:** Jing Zhao, Xilong Chen, Shixia Yue

**Affiliations:** Gansu Provincial Maternity and Child-care Hospital (Gansu Provincial Central Hospital), Lanzhou, China

**Keywords:** hemophagocytic lymphohistiocytosis (HLH), Epstein–Barr virus (EBV) infection, NBAS gene variant, gene mutation, bad prognosis

## Abstract

Hemophagocytic syndrome (HPS), also known as hemophagocytic lymphohistiocytosis (HLH), is a life-threatening disorder that can occur in both children and adults, closely associated with genetic factors and acquired immune dysregulation. This article presents a case report of HLH with NBAS gene mutation and chronic active Epstein–Barr virus (EBV) infection. Despite undergoing a series of aggressive treatments, the patient failed to achieve a favorable clinical response. The clinical course was complicated by hepatic injury, coagulopathy, progressive cytopenia in all three blood cell lineages, and significant elevation of serum ferritin, ultimately resulting in fatal outcome. Current research on genetic predisposing factors has identified 17 causative genes for HLH, including PRF1 and UNC13D. However, NBAS-related cases have been rarely reported. The discovery of additional potential pathogenic genes holds significant value for advancing diagnostic and therapeutic approaches in HLH management.

## Introduction

HLH is a rare and highly fatal clinical disorder affecting both children and adults, characterized by persistent yet ineffective immune activation triggered by primary or secondary factors, leading to excessive systemic inflammatory responses. Clinical manifestations may include fever, hepatosplenomegaly, pancytopenia, coagulopathy, hypertriglyceridemia, elevated serum ferritin, and hemophagocytosis identified in bone marrow, spleen, or lymph node biopsies. The disease was first described in 1939 by Scott and Robb-Smith as “histiocytic medullary reticulosis” (1). Familial features of HLH were identified by Farquhar and Claireaux, who first reported its genetic pathogenicity (2).

Studies indicate that most pediatric HLH cases involve genetic defects, primarily homozygous or compound heterozygous mutations inherited in a Mendelian pattern (3). Genes such as PRF1, UNC13D, STX11, and STXBP2 have been definitively linked to HLH pathogenesis. These genes encode perforin, Munc13-4, syntaxin 11, and syntaxin-binding protein 2, respectively—proteins critical for lymphocyte cytotoxic activity. Deficiencies in these proteins impair the function of natural killer (NK) cells and cytotoxic T lymphocytes (CTLs), which are central to disease development (4).

In many pediatric patients, genetic mutations coexist with secondary factors. Infections, for example, may act as triggering events in most cases, while underlying genetic defects dictate disease progression and poor prognosis. NBAS encodes a protein with two leucine zipper domains, a ribosomal protein S14 signature domain and a Sec39 like domain. The protein is thought to be involved in Golgi-to-ER transport. Mutations in this gene are associated with short stature, optic nerve atrophy, and Pelger-Huet anomaly.

In a study by Xiaoman Bi et al., whole-genome/exome sequencing of 13 families with HLH-affected children identified NBAS variants in 2 cases. Validation in a cohort of 224 HLH patients revealed an NBAS variant frequency of 2.11%. Using lentiviral transfection to knock down NBAS expression in cellular models, flow cytometry analysis demonstrated significantly reduced cytotoxic activity and degranulation capacity compared to controls (5), partially elucidating the mechanistic role of this gene in HLH pathogenesis.

## Case presentation

**Figure 1:**
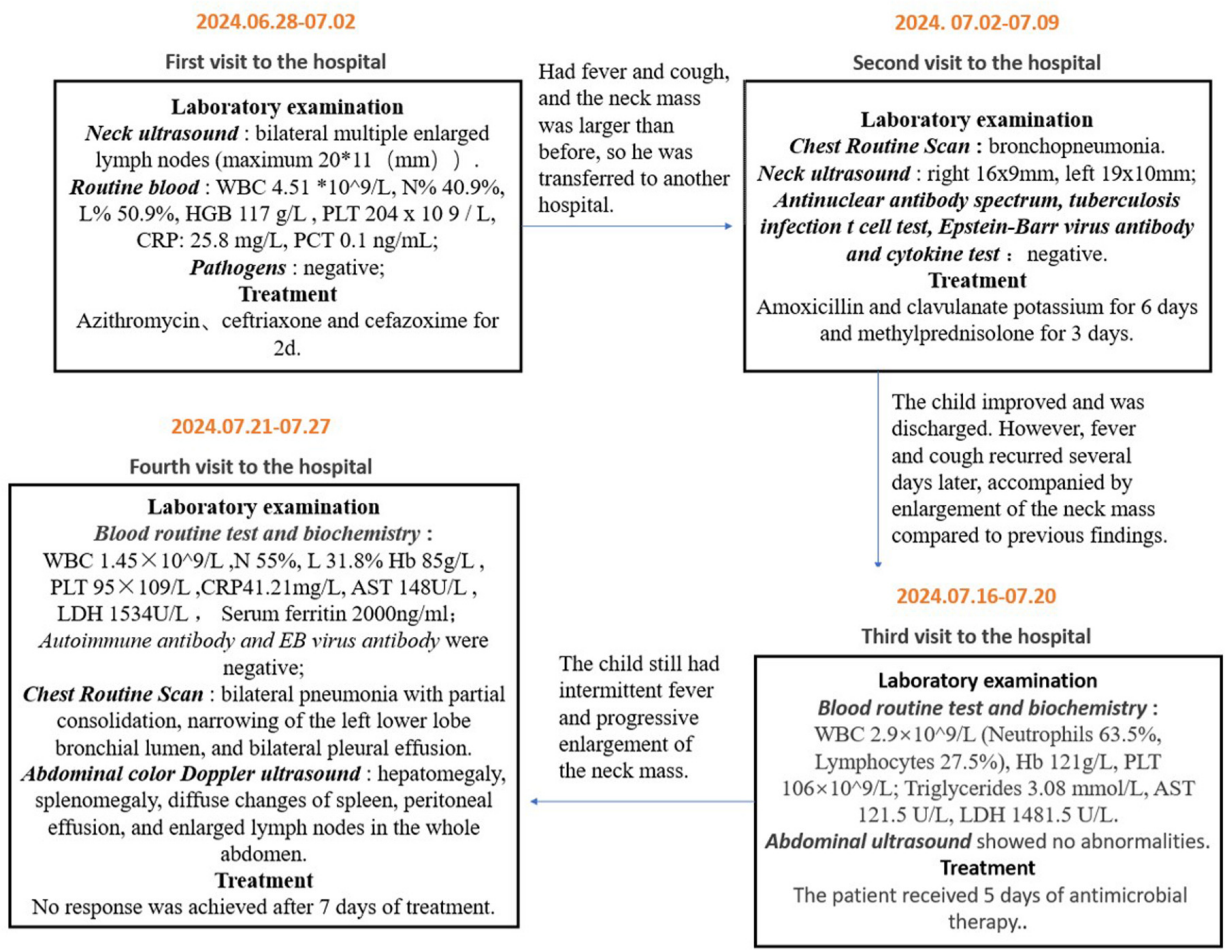
Pre-admission clinical timeline (including diagnostic workup, therapies, and outcomes).

A 3-year-and-7-month-old boy of Hui ethnicity was admitted to the hospital with the chief complaint of “intermittent fever accompanied by neck mass for over one month”. Prior to admission, he had experienced recurrent fever for over a month, with a peak temperature of 40°C. Despite treatment with physical cooling measures and oral antipyretics, his fever persisted. Associated symptoms included cough with sputum production, but no nausea, vomiting, diarrhea, chills, convulsions, or coma. He had sought medical care at multiple local hospitals, with details of prior visits summarized in Figure 1. Upon readmission, he continued to exhibit fever without significant reduction in peak temperature, though his cough and sputum production had improved.

Physical examination on admission revealed an alert but lethargic child. No scattered rash was observed. The pharynx was erythematous, with non-enlarged bilateral tonsils. A mobile, soybean-sized lymph node with firm consistency and no tenderness was palpated in the left neck. The right neck exhibited significant egg-sized swelling with ulceration (no purulent discharge) and normal skin temperature. No other positive signs were noted. Past medical history, family history, and personal history were unremarkable.

Upon admission, comprehensive auxiliary examinations were performed, for example, complete blood count (CBC), biochemical tests, coagulation tests, serum ferritin, NK cell activity assay, and sCD25 detection, leading to a preliminary diagnosis of HLH. Multiple EB virus nucleic acid tests conducted both pre-admission and post-admission returned negative results, leaving the etiology undetermined. In accordance with the Diagnosis and Treatment Guidelines for Hemophagocytic Syndrome (1994 and 2004 editions), symptomatic and supportive therapies were initiated, including ceftazidime for anti-infection, etoposide for chemotherapy, methylprednisolone sodium succinate to suppress inflammatory immune responses, and human immunoglobulin for immune modulation.

After one day of treatment, the child's fever subsided with general improvement, though cervical lymphadenopathy persisted, particularly on the right side. Following two weeks of standardized chemotherapy, re-evaluation showed a significant decline in serum ferritin levels but marked bone marrow suppression and agranulocytosis. Poor healing of the ulcerated cervical lymph nodes suggested suboptimal efficacy of etoposide. Literature review prompted a switch to ruxolitinib for an additional 10 days, yet therapeutic outcomes remained unsatisfactory.

**Figure 2:**
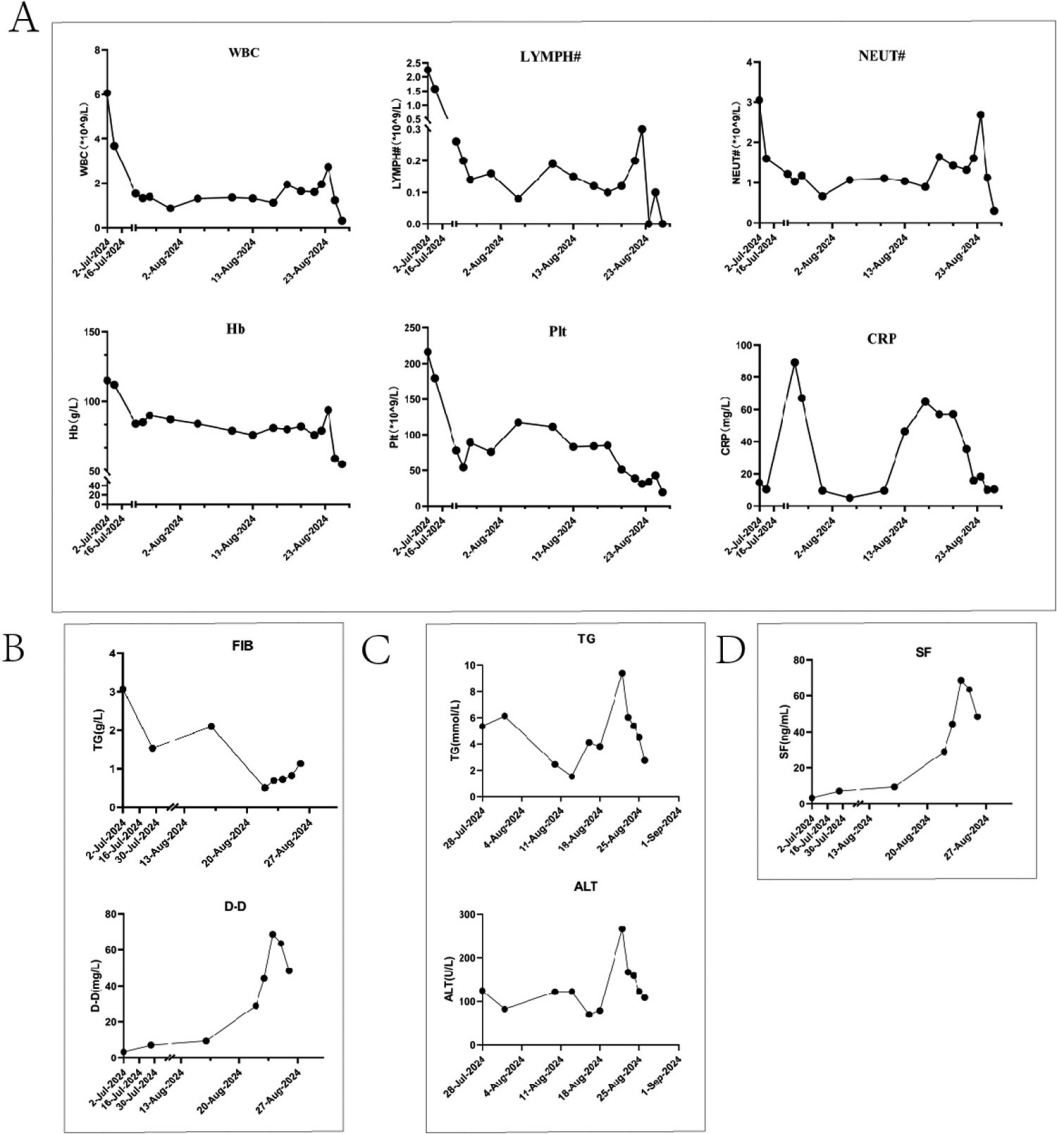
Illustrates the dynamic trends of key parameters during the diagnostic and therapeutic course. **(A)** Displays the trends of white blood cell count (WBC), hemoglobin, platelet count, C-reactive protein (CRP), absolute lymphocyte count, and absolute neutrophil count. **(B)** Depicts the changes in coagulation parameters (D-dimer and fibrinogen). **(C)** Shows the variations in alanine aminotransferase (ALT) and triglyceride levels. **(D)** Highlights the dynamic trajectory of serum ferritin.

Subsequent worsening of right cervical swelling, confirmed by neck ultrasound, revealed progression toward the head and midline neck with tracheal compression. Concurrently, the child developed escalating fever and reduced SpO2 on arterial blood gas analysis, necessitating immediate invasive mechanical ventilation. It was considered that the child may still have infection, further investigations were pursued, including Brucella blood testing, blood pathogen metagenomic sequencing, and cervical lymph biopsy. Results later identified EB virus positivity and chronic active EBV infection, prompting initiation of ganciclovir antiviral therapy. During this period, the child exhibited progressive hepatic dysfunction. Plasma exchange and cytokine adsorption were performed to eliminate inflammatory mediators. A multidisciplinary team revised the chemotherapy regimen to etoposide, ruxolitinib, cyclosporine, and dexamethasone. However, no clinical improvement was observed, with persistent deterioration in liver function, coagulation parameters, and ventilator weaning failure. Despite transfusions and repeated plasma exchange, therapeutic responses were negligible. After full disclosure of the prognosis, the family opted to withdraw care. The child was discharged with nasal cannula oxygen support. Post-discharge whole-exome sequencing identified a heterozygous NBAS gene mutation. Our genetic testing report confirmed this variant was paternally inherited, with the mother showing wild-type status. Follow-up confirmed the child's death one day after discharge. Dynamic changes in laboratory parameters throughout the treatment course are illustrated in Figure 2.

## Discussion

This article reports a case of a child with NBAS gene variant combined with Epstein–Barr virus (EBV)-associated hemophagocytic lymphohistiocytosis (HLH). EBV infection is one of the most common triggers of secondary HLH, and its mechanism may involve virus-mediated hyperactivation of the immune system. EBV can infect lymphoid and myeloid cells (6), triggering a cytokine storm that leads to abnormal macrophage activation, phagocytosis of blood cells, and ultimately multi-organ dysfunction. The patient presented with recurrent high fever, trilineage cytopenia, and markedly elevated ferritin (7), meeting the HLH-2004 diagnostic criteria. Initial repeated EBV antibody and nucleic acid tests were negative; however, blood next-generation sequencing (NGS) and cervical lymph node biopsy later confirmed EBV infection, supporting EBV as the direct trigger of HLH in this case.

The NBAS gene encodes a protein involved in Golgi-to-endoplasmic reticulum (ER) retrograde transport. Its variants are associated with multiple disorders, including infantile liver failure syndrome (ILFS2) (8–10) and immune dysregulation (11, 12). Literature review reveals rare reports directly linking NBAS to HLH. Studies by Xiaoman Bi et al. suggest that among known HLH-associated genes, NBAS ranks as the second most frequently mutated gene (2.11%) after PRF1. Germline defects in 12 known HLH genes impair cytotoxic degranulation of natural killer (NK) cells or cytotoxic T lymphocytes (CTLs), driving HLH pathogenesis. NBAS, functioning upstream in the degranulation pathway, participates in retrograde transport from the Golgi to the ER. NBAS-deficient NK cell lines exhibit impaired cytotoxicity and degranulation (5). This case highlights delayed diagnosis due to absent EBV-specific antibody responses post-infection. Beyond gene-environment interactions, NBAS defects may also dysregulate immune and metabolic pathways, exacerbating disease progression.

Current understanding of the mechanism linking NBAS variants and EBV-associated HLH remains limited. We hypothesize that NBAS variants may disrupt ER stress responses, impairing immune cell (e.g., T/NK cell) function (13) and amplifying aberrant immune activation post-EBV infection. The ER, critical for protein synthesis, folding, and modification, may—when dysfunctional—compromise protein quality/quantity, disrupting cellular metabolism and increasing tissue injury risk, aligning with HLH features such as elevated liver enzymes and coagulopathy. Additionally, NBAS heterozygosity may act as a genetic susceptibility factor, heightening HLH risk under the “second hit” of EBV infection, akin to genetic backgrounds in other secondary HLH cases. These hypotheses underscore a potential NBAS-EBV-HLH nexus. Future studies using animal models or *in vitro* systems are needed to validate NBAS's mechanistic role in HLH.

EBV-HLH manifests with diverse, nonspecific clinical features, necessitating comprehensive evaluation of laboratory findings and bone marrow hemophagocytosis. While this patient exhibited classic HLH signs, initial negative EBV nucleic acid and antibody tests delayed identification of the secondary trigger. Thus, genetic testing is critical in suspected HLH cases lacking typical features or clear etiology. Despite guideline-directed therapy (e.g., etoposide, ruxolitinib, cyclosporine, dexamethasone), the patient showed poor response. Notably, NBAS variants may alter drug metabolism or increase hepatotoxicity risk. Persistent deterioration of liver and coagulation function during later stages may reflect genetic influences. Given medical constraints and family preferences, hematopoietic stem cell transplantation was deemed unfeasible, and palliative care was chosen. The fatal outcome aligns with the high mortality of EBV-HLH and suggests genetic variants may portend poorer prognosis.

Prior studies have identified multiple genes critical to HLH pathogenesis, yet many mechanisms remain undiscovered. Expanding genetic testing (including non-classical HLH-associated genes like NBAS) in refractory or recurrent cases could uncover hidden genetic contributors. For HLH patients with confirmed variants, targeted immunomodulatory or gene-specific therapies may improve outcomes. Future research should focus on elucidating these mechanisms to refine therapeutic strategies.

## Conclusion

This case report describes a child with EBV-associated HLH and an NBAS gene variant, expanding our understanding of the genetic landscape of HLH. Despite aggressive treatment, the outcome was poor, reflecting the high mortality and dismal prognosis of such diseases, particularly when compounded by genetic variants. The NBAS variant may exacerbate post-EBV inflammatory responses via immune-metabolic pathways, underscoring the need to investigate non-classical genes in secondary HLH. Future efforts should focus on accumulating more cases and functional studies to clarify underlying mechanisms and refine therapeutic strategies.

## Data Availability

The raw data supporting the conclusions of this article will be made available by the authors, without undue reservation.
